# The 8-bromobaicalein inhibited the replication of dengue, and Zika viruses and targeted the dengue polymerase

**DOI:** 10.1038/s41598-023-32049-x

**Published:** 2023-03-25

**Authors:** Siwaporn Boonyasuppayakorn, Thanaphon Saelee, Thao Nguyen Thanh Huynh, Rita Hairani, Kowit Hengphasatporn, Naphat Loeanurit, Van Cao, Vipanee Vibulakhaophan, Panattida Siripitakpong, Parveen Kaur, Justin Jang Hann Chu, Chairat Tunghirun, Opas Choksupmanee, Sarin Chimnaronk, Yasuteru Shigeta, Thanyada Rungrotmongkol, Warinthorn Chavasiri

**Affiliations:** 1 Center of Excellence in Applied Medical Virology, Department of Microbiology, Faculty of Medicine, Chulalongkorn University, Bangkok, 10330 Thailand; 2 Center of Excellence in Natural Products Chemistry, Department of Chemistry, Faculty of Science, Chulalongkorn University, Bangkok, 10330 Thailand; 3 Center for Computational Sciences, University of Tsukuba, 1-1-1 Tennodai, Tsukuba, Ibaraki 305-8577 Japan; 4 Graduate School, Interdisciplinary Program in Microbiology, Chulalongkorn University, Bangkok, 10330 Thailand; 5 Department of Biology, Faculty of Science, Chulalongkorn University, Bangkok, 10330 Thailand; 6 Center of Excellence in Biocatalyst and Sustainable Biotechnology, Department of Biochemistry, Faculty of Science, Chulalongkorn University, Bangkok, 10330 Thailand; 7 Department of Microbiology and Immunology, Yong Loo Lin School of Medicine, National University of Singapore, Singapore, 117545 Singapore; 8 Infectious Diseases Translational Research Programme, Yong Loo Lin School of Medicine, National University of Singapore, Singapore, Singapore; 9 NUS Medicine BSL3 Core Facility, Yong Loo Lin School of Medicine, National University of Singapore, Singapore, Singapore; 10 Institute of Molecular and Cell Biology (IMCB), A*STAR, Singapore, Singapore; 11 The Laboratory of RNA Biology, Institute of Molecular Biosciences, Mahidol University, Salaya Campus, Nakhon Pathom, 73170 Thailand; 12 Program in Bioinformatics and Computational Biology, Faculty of Science, Chulalongkorn University, Bangkok, 10330 Thailand

**Keywords:** Drug discovery, Microbiology, Molecular biology

## Abstract

Dengue and Zika viruses are mosquito-borne flaviviruses burdening millions every year with hemorrhagic fever and neurological symptoms. Baicalein was previously reported as a potential anti-flaviviral candidate and halogenation of flavones and flavanones potentiated their antiviral efficacies. Here, we reported that a chemically modified 8-bromobaicalein effectively inhibited all dengue serotypes and Zika viruses at 0.66–0.88 micromolar in cell-based system. The compound bound to dengue serotype 2 conserved pocket and inhibited the dengue RdRp activity with 6.93 fold more than the original baicalein. Moreover, the compound was mildly toxic against infant and adult C57BL/6 mice despite administering continuously for 7 days. Therefore, the 8-bromobaicalein should be investigated further in pharmacokinetics and efficacy in an animal model.

## Introduction

Dengue, and Zika viruses share the same geographical niche as they are transmitted by the same mosquito, *Aedes aegypti*^1^. Dengue has placed 2 billion people at risk every year and hospitalized about 500,000 people with severe hemorrhage, shock, and multiple organ failure^2,3^. There are four serotypes of dengue virus (DENV1-4) with a minimum of 67% amino acid sequence identity among all DENV serotypes^4^. The secondary heterotypic infection misleads the immunological response towards the prior infection^5,6^. Macrophages opsonize partially neutralized viruses, thus multiplying the viral replication and increasing severity^7^. Targeting drugs towards viral replication protects the infected individuals from severe progression in the critical phase^8^. Zika virus (ZIKV) is recently known for in utero transmission, causing microcephaly in newborns in the non-immune population^9^. Both dengue and Zika viruses are members of the family *Flaviviridae*. Zika and dengue RdRp sequence identity is 68.6%, and the similarity is 79.2%^10^, and their antibodies cross-reacted in plaque-reduction neutralization assay^11,12^. Therefore, anti-dengue leads should also effectively inhibit Zika virus replication.

Flavones and flavanones are potential anti-flaviviral leads^13–19^. Moreover, addition of a Bromine to the flavone and flavanones increase the antiviral efficacies^20,21^, reduce cytotoxicities, and increase aqueous solubility^22^. Among the flavone and flavanones, baicalein and its glycosylated derivative, baicalin, were the most potent inhibitors of dengue^19,23^ and SARS-CoV-2^24^. Therefore, adding a Br to the baicalein could increase the potency of the antiviral effect. In this study, we synthesized an 8-bromobaicalein and characterized it as a potential lead against dengue, and Zika viruses. Moreover, we identified the viral RNA-dependent RNA polymerase as one of the potential targets by integrating computational, enzyme-based, and cell-based methods. The compound was not toxic in various cell lines and in a mouse model.

## Results

**Figure 1:**
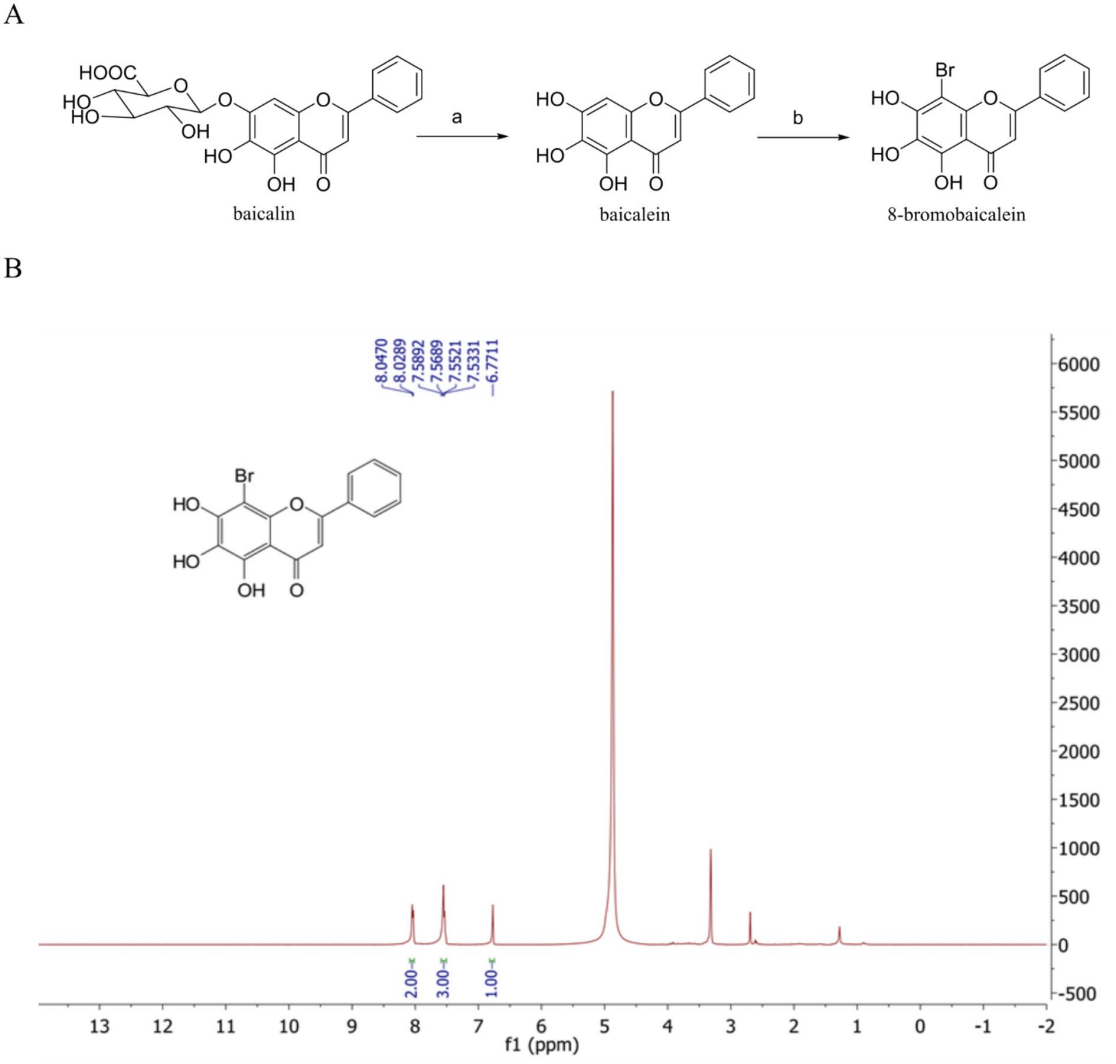
(**A**) Synthesis of an 8-bromobaicalein in a two-step process and (**B**) identification by ^1^H-NMR (CD_3_OD, 400 MHz).

The 8-bromobaicalein was synthesized in a two-step process (Fig. 1A), including hydrolysis of baicalin^25^ and bromination of baicalein^26^. The bromination occurred exclusively at the 8-position of the C-ring. The identity was determined using ^1^H-NMR(CD_3_OD, 400 MHz) (Fig. 1B) and 13C-NMR (Supplementary Fig. S1). The newly synthesized 8-bromobaicalein was first tested for cytotoxicities in various cell lines (Table 1, Supplementary Fig. S2). The compound was not toxic to all tested cells except for the Huh-7 that showed mild toxicity at 97.89 ± 7.06 µM. Moreover, 8-bromobaicalein also showed submicromolar level of efficacies against DENV1-4, and ZIKV (Table 2, Supplementary Fig. S3), relatively more potent than the original baicalein (Table 2, Supplementary Fig. S4). Similarly, the parent compound, baicalein, was not cytotoxic to the LLC/MK2 with the CC_50_ of > 250 µM (Table 1, Supplementary Fig. S4). The selectivity index (SI), or the ratio between cytotoxicity and antiviral activity of the same cell line (LLC/MK2), determined the potential therapeutic range of the compound. In this case, the 8-bromobaicalein exhibited SIs at about 300 in dengue and Zika viruses allowing the broader range for drug administration. However, the compound did not inhibit the nonenveloped enterovirus-A71 causing hand-foot-mouth disease (Supplementary Fig. S3). Therefore, this compound was a promising lead against mosquito-borne flaviviruses.

**Table 1:** Cytotoxicity of 8-bromobaicalein to cell lines. An error was calculated from three independent experiments.

Cells	8-bromobaicalein CC_50_ (µM)
THP-1	> 100
HepG2	> 100
HEK	> 100
LLC/MK2	> 250 (baicalein CC_50_ > 250)
Huh7	97.89 ± 7.06
RD	> 100

**Table 2:** Efficacy of an 8-bromobaicalein to dengue, zika and enteroviruses in LLC/MK2 cells.

No.	Virus (strain)	8-bromobaicalein EC_50_ (µM)	Selectivity index (SI)	baicalein EC_50_ (µM)	Selectivity index (SI)
1.	DENV1 (16008)	0.66 ± 0.05	> 378.7879	1.40 ± 0.38	> 178.57
2.	DENV2 (NGC)	0.88 ± 0.14	> 284.0909	4.04 ± 1.37	> 61.88
3.	DENV3 (16562)	0.80 ± 0.08	> 312.5	1.19 ± 0.66	> 210.08
4.	DENV4 (1036)	0.80 ± 0.15	> 312.5	0.93 ± 0.13	> 287.35
5.	ZIKV (SV0127/14)	0.72 ± 0.20	> 347.2222	1.58 ± 0.42	> 158.23
6.	EV-A71 (BRCR)	> 25	NA	NA	NA

Errors were calculated from three independent experiments.

*NA* (not applicable) refers to the experiments not performed.

No statistical significance was found between the results of both compounds (unpaired t-test).

**Figure 2:**
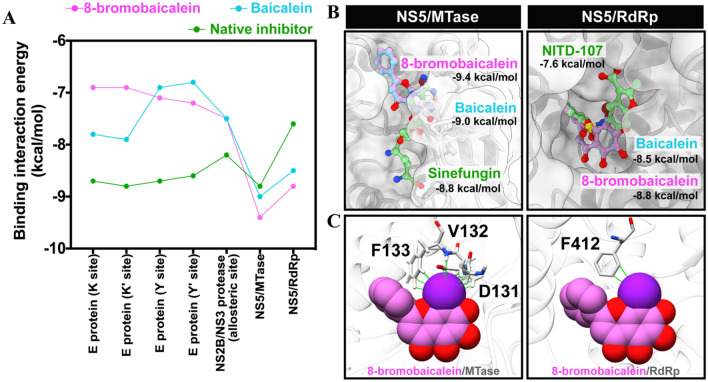
(**A**) Binding interaction energy (kcal/mol) of baicaleins with the four targets relative to known inhibitors. (**B**) Ligand conformation and (**C**) halogen interaction at the MTase and RdRp binding sites.

The molecular target of the focused compounds was primarily explored using molecular docking. The 8-bromobaicalein and baicalein were individually docked against dengue envelope (E) protein, nonstructural protein 2B/3 (NS2B/NS3) protease, methyltransferase (NS5/MTase), and RNA-dependent RNA polymerase (NS5/RdRp) using Autodock VinaXB (Fig. 2A) The 8-bromobaicalein showed a higher binding affinity than its parent compound baicalein in NS5/MTase (− 9.4 and − 9.0 kcal/mol) and NS5/RdRp (− 8.8 and − 8.5 kcal/mol) and even better than the native inhibitors sinefungin and NITD107, accordingly. These two baicaleins demonstrated similar binding patterns in the SAM-binding and RdRp pockets of the NS5/MTase and NS5/RdRp domains, respectively (Supplementary Figs. S5, S6). As of the NS5/MTase, the baicaleins were stabilized by the three hydrogen bonding residues T104, K105, and H110 at the SAM-binding pocket, whereas more residue contributions (S56, R84, G85, G86, K105, H110, D131, V132, and D146) were found for sinefungin binding (Supplementary Fig. S5). Baicaleins’ B-ring core structure and sinefungin’s imidazole ring were similarly aligned into the pocket and shared the two common interacting residues, K105 and H110.

Moreover, a similar orientation was observed in the flavonoid B-ring and the NITD chlorobenzene ring aligned in the NS5/RdRp pocket. Baicaleins generated three hydrogen bonds with N492 and the two conserved residues Q602 and G604^27,28^, whereas NITD-107 formed two hydrogen bonds with only a T413 residue (Supplementary Fig. S6). It is noted that the bromine substitution showed interactions with the NS5/MTase residues D131, V132, and F133, and the NS5/RdRp residue F412 (Fig. 2C), corresponding to the stronger affinity of 8-bromobaicalein. Consequently, molecular dynamics (MD) simulations of baicaleins in complex with these two possible targets were performed in an aqueous solution.

**Figure 3:**
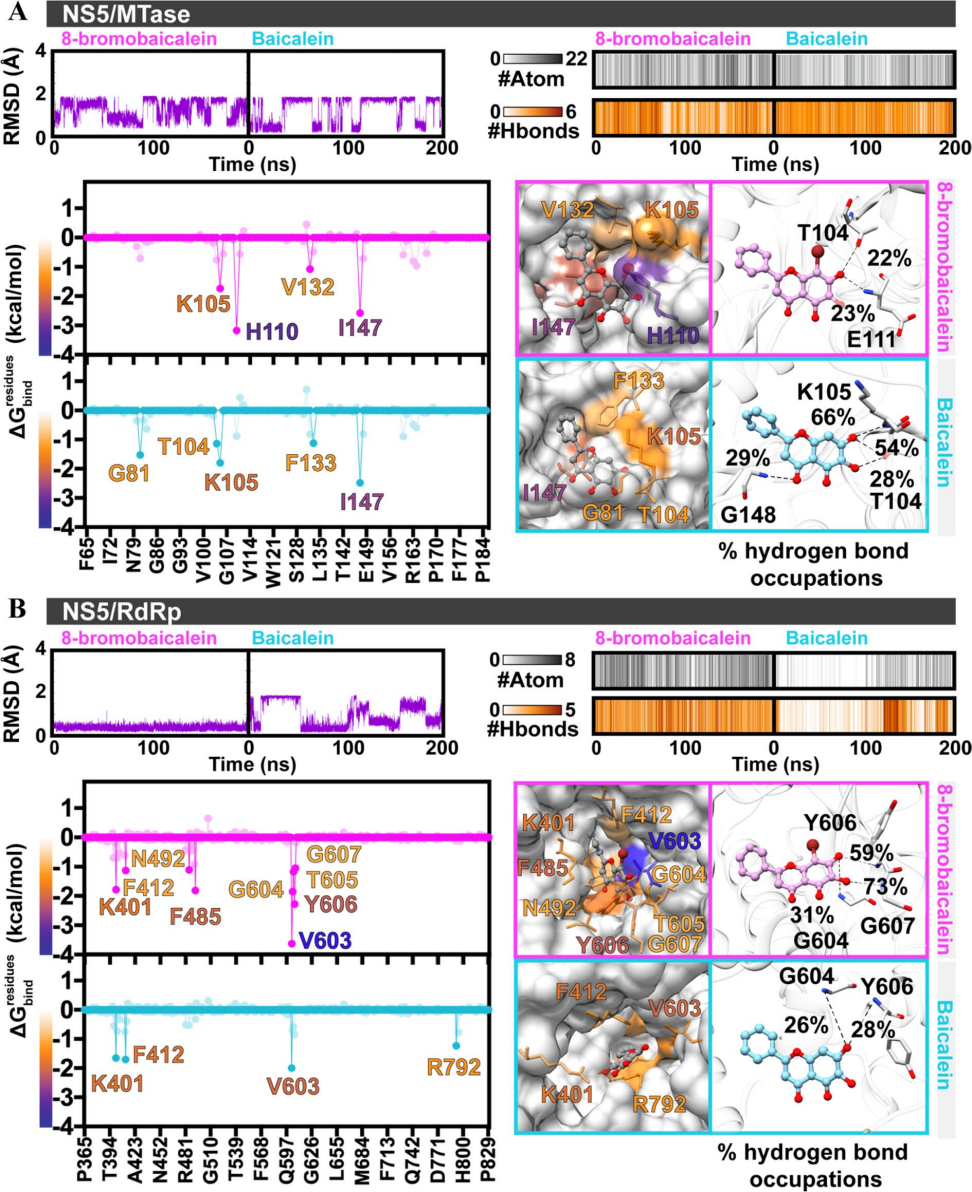
RMSD of ligand atoms, atom contacts (#Atom), number of intermolecular hydrogen bonds (#H-bond), per-residue decomposition free energy ($\Delta {\text{G}}_{{{\text{bind}}}}^{{{\text{residue}}}}$) and H-bond occupation (> 20%) for (**A**) baicaleins/MTase and (**B**) baicaleins/RdRp.

From the 200-ns MD simulations, ligand’s RMSD value, the number of intermolecular hydrogen bonds, the number of atom contacts, and the per-residue decomposition energy ($\Delta {\text{G}}_{{{\text{bind}}}}^{{{\text{residue}}}}$) calculated from the last 50-ns snapshots were illustrated in Fig. 3. The RMSD results revealed a constantly changing orientations of 8-bromobaicalein and baicalein in the SAM-binding pocket of MTase and the baicalein in the RdRp binding site along with simulation time, suggesting unstable interactions (Fig. 3A,B). Only the 8-bromobaicalein was stable in RdRp binding site (< 0.4 Å) in RMSD (Fig. 3B). This finding was well supported by more hydrogen bonds and atom contacts (Fig. 3A,B) as well as residue stabilizations (K401, F412, F485, N492, V603, G604, T605, Y606, and G607 in Fig. 3B) contributed for 8-bromobaicalein binding to RdRp. The introduction of bromine could enhance the cumulative binding affinity especially from the adjacent molecules, especially at the V603, G604, T605, Y606, and G607 (Fig. 3B).

In addition, the susceptibility of baicaleins in complex with NS5/RdRp was confirmed by the MM/GBSA binding free energy calculation (Fig. 3A,B). The result showed that the binding strength of 8-bromobaicalein (− 3.93 ± 1.14 kcal/mol) was stronger than that of baicalein (3.44 ± 1.60 kcal/mol). All obtained results from in silico study recommended that NS5/RdRp was more likely be the potential target for 8-bromobaicalein.

**Figure 4:**
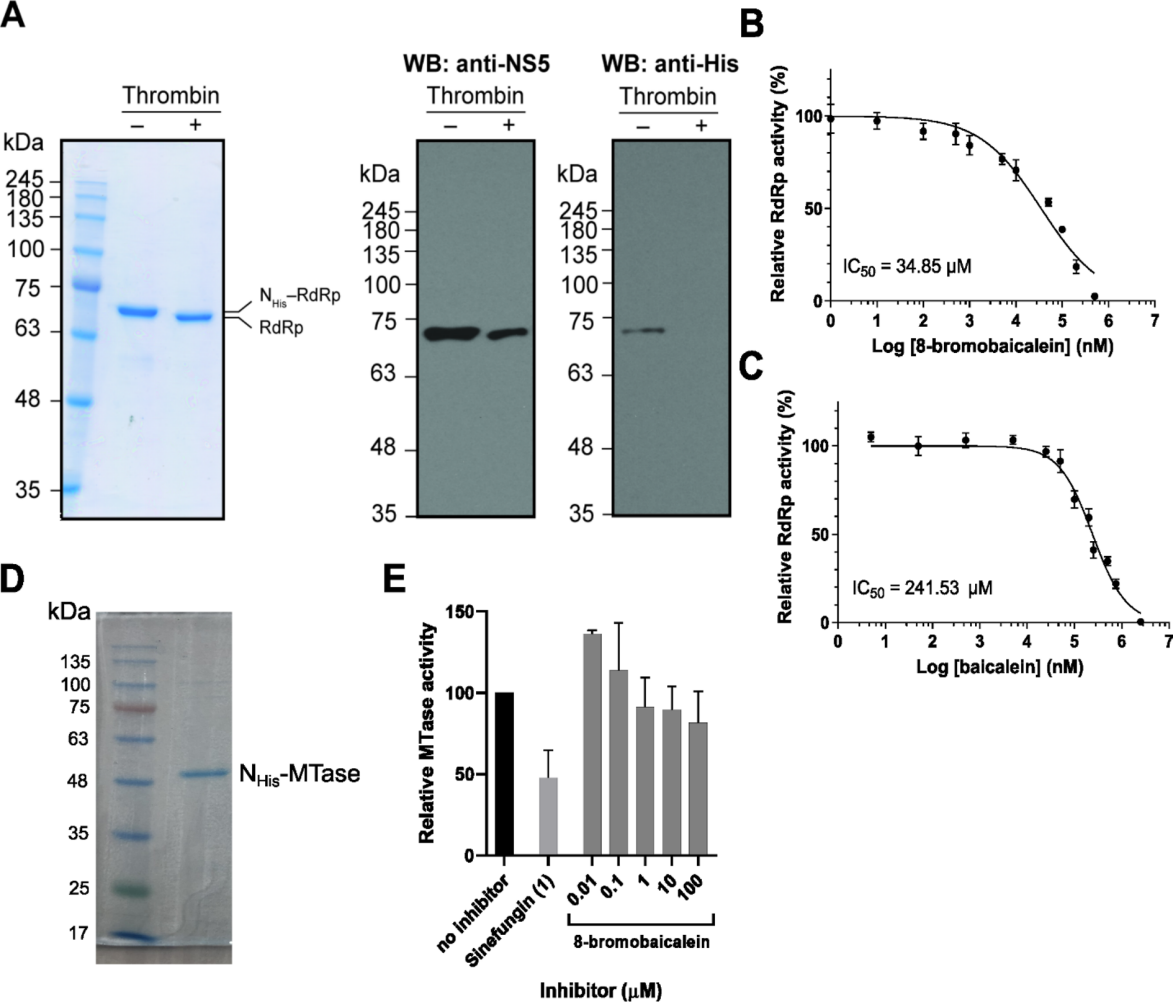
(**A**) Coomassie staining (upper panel) and western blotting (lower panel) of purified RdRps upon thrombin treatment. (**B**) IC_50_ of 8-bromobaicalein and (**C**) baicalein was determined by the RdRp assay. (**D**) Coomassie staining of purified MTase and (**E**) its activities in the presence of 8-bromobaicalein.

The in vitro RNA-dependent RNA polymerase and methyltransferase assays were used to confirm it as a target of the 8-bromobaicalein (Fig. 4). The NHis-RdRp was adequately expressed in a soluble form with molecular size of 75 kDa and purified by a high affinity between His-tag and nickel ion. The His-tag removal was essential to improve the polymerase activity of DENV2 NS5/RdRp^29^. Thrombin protease was directly subjected to IMAC elution fraction during dialysis. The low salt buffer was used in dialysis in order to prevent protein precipitation. The 73.2 kDa of non-tagged NS5/RdRp was successfully purified by size exclusion chromatography and showed over 95% purity as assessed by Coomassie staining (Fig. 4A, upper panel). Moreover, the protein expression and tag removal were confirmed using western blotting against anti-NS5 and anti-His antibodies. A successful tag-removal using thrombin treatment was established as the signal of RdRp, but not His-tag, was observed (Fig. 4A, lower panel). The 8-bromobaicalein inhibited the RdRp activity with the IC_50_ of 34.85 µM (Fig. 4B), whereas the IC_50_ of baicalein was 241.53 µM (Fig. 4C). The result suggested that RdRp was the most likely a target of 8-bromobaicalein. Moreover, the 8-bromobaicalein was tested for inhibition of methyltransferase activity. The MTase was expressed, purified^30^ (Fig. 4D) and the activity was measured using MTaseGlo assay. Sinefungin at 1 µM was used as a positive control. Results showed that the 100 µM compound could not reach the 50% MTase inhibition (Fig. 4E). Therefore, we concluded that the RdRp, not MTase, was the most likely a target of 8-bromobaicalein.

**Figure 5:**
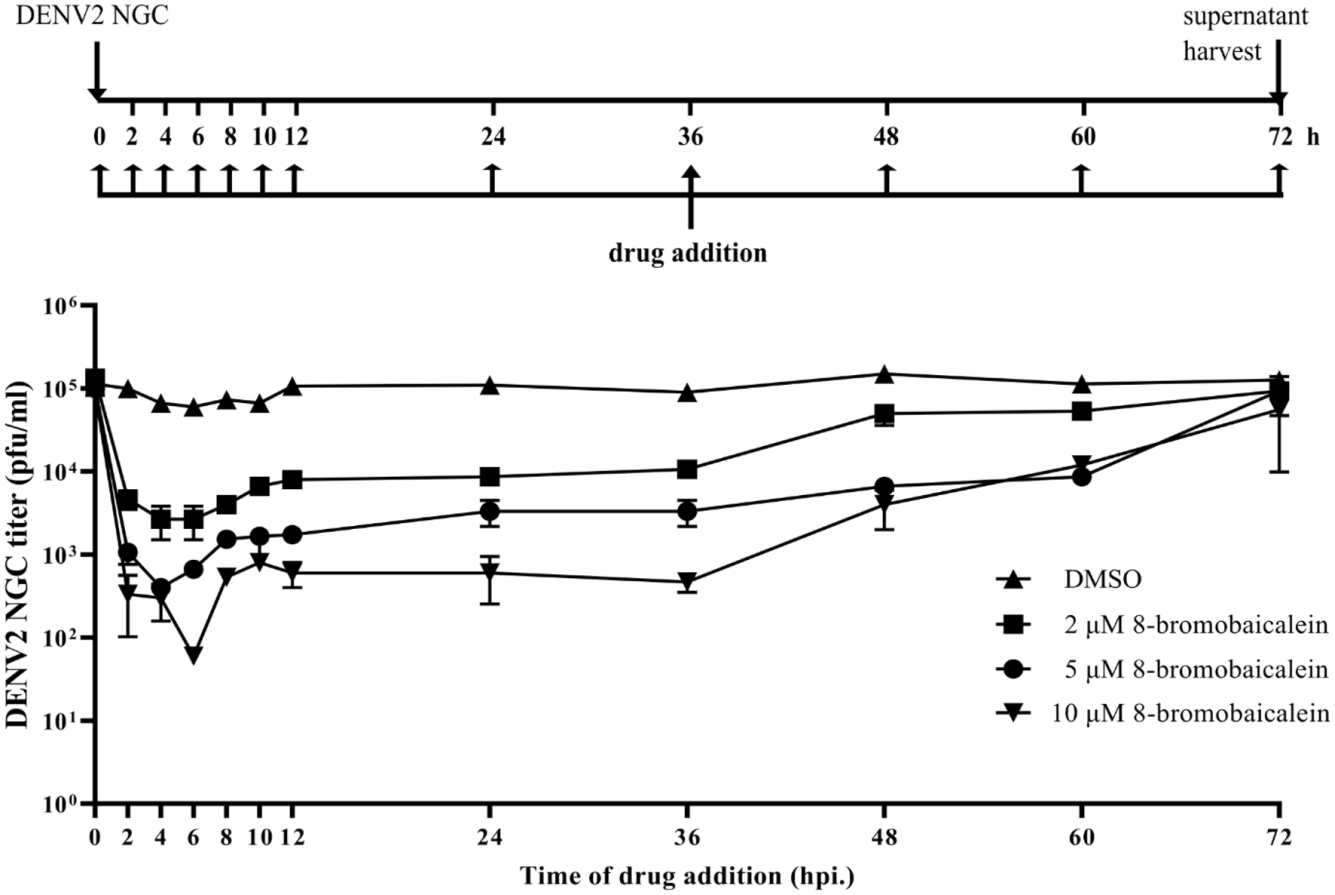
A scheme and DENV2 titers (pfu/ml) in LLC/MK2 cells of time-of-drug addition study. Briefly, the 2, 5, and 10 µM 8-bromobaicalein was added into DENV2-infected cells (M.O.I. of 0.1) at various time points. All supermatants were harvested at 72 h and quantified by plaque assay. Errors indicated the standard deviation of three biological replicates.

The cell-based time-of-addition study was performed with 2, 5, or 10 µM of 8-bromobaicalein at various time points after infection (Fig. 5). The compound consistently inhibited the virion progeny by 1–2.5 log concentrations (pfu/mL) in a dose-dependent manner when added within 12 h after infection. The potency gradually declined at 48 h and later time-points but the dose-dependent pattern was still retained. c The results were consistent with the previous computational and enzymatic assays (Figs. 2, 3, 4).

**Figure 6:**
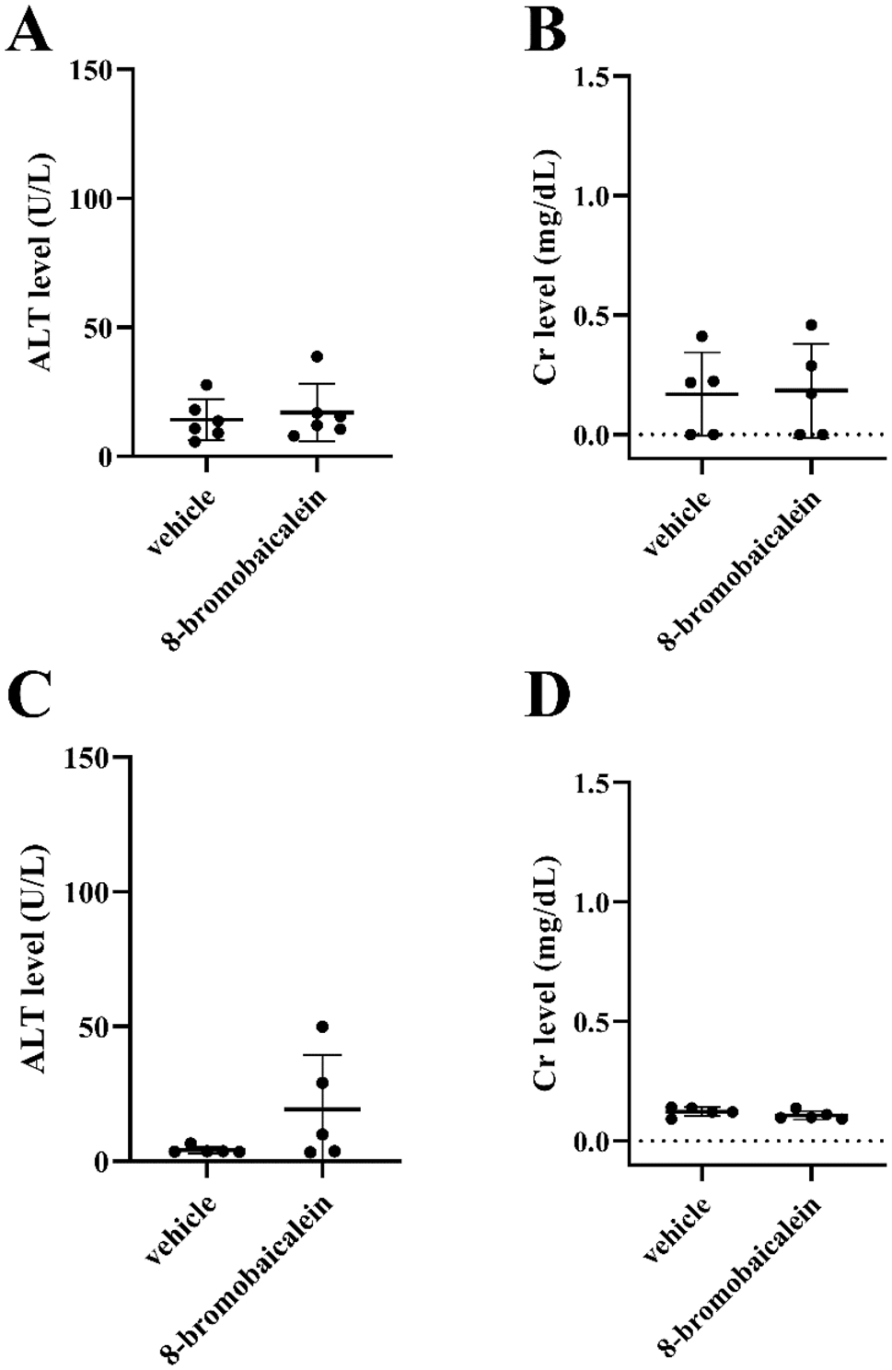
Animal Toxicity. (**A**) ALT and (**B**) Cr levels in infant mice and (**C**) ALT and (**D**) Cr levels in adult mice taken at the 8^th^ day after seven consecutive days of 10 mg/kg 8-bromobaicalein, once daily, intraperitoneal administration. Plasma samples were collected for analysis of hepatorenal functions. Each data point represented the ALT or Cr levels of each individual. Error bars indicated the standard deviation of the means. No significant differences between the two populations were found (unpaired t-test).

C57BL/6 mice were used to evaluate the compound toxicity according to previous description^22^. The compound at 10 mg/kg was administered intraperitoneally into both infant and adult mice once daily for consecutive 7 days (Fig. 6). The intraperitoneal route was chosen to avoid the first pass effect that metabolized at least 90% of the flavonoids. The 10 mg/kg concentration was selected according to the previous pharmacokinetic study of the original baicalein^31^. All mice were healthy throughout the experiments. The clinical scorings were also within normal range. Alanine transaminase (ALT) and plasma creatinine (Cr) levels at the 8th day after the first injection were insignificantly different in treated and control groups (Fig. 6A–D). Noted that the treated group exhibited a slight ALT increase only in the adult cohort (Fig. 6C). In summary, the chemically modified 8-bromobaicalein at the concentration of 10 mg/kg did not show any significant hepatorenal toxicity to immunocompetent mice.

## Discussion

Flavones and flavanones were broad-spectrum antivirals. Baicalein (5,6,7-trihydroxyflavone) exhibited an antiviral effect with the IC_50_ of 1.55 μg/mL or 5.74 µM against the DENV2^23^, and Japanese encephalitis virus (JEV)^32^. Moreover, a glycoside derivative, baicalin, showed a broader array of viral inhibitions, including DENV^19^, CHIKV^33^, herpes simplex virus^34^, and influenza A viruses^35^. The original baicalein was also mildly toxic with the CC_50_ of > 250 µM in LLC/MK2 cells, > 100 µM in Vero^23,32^, > 50 µM in A549^36^, and > 200 µM in HaCat cells^34^. Moreover, the original baicalein showed neuroprotective^37^, hepatoprotective^38^, and anti-inflammation^39^ effects. Therefore, baicalein was chosen as a core structure for a modification.

Structural modification potentiates its function by increasing efficacies and aqueous solubility while preserving the cytoprotective effect. According to previous reports, halogenation was a method of choice for a chemical modification with flavone^21^, flavanones^40^, and dihydrorugosaflavonoids^41^ as halogenated flavonoids increased the efficacies by 10–30 folds against DENV or CHIKV replication. The 8-bromobaicalein was synthesized under a two-step process and identified by the ^1^H-NMR (Fig. 1). The compound was not toxic to most cells at 100 µM (Table 1, Supplementary Fig. S2), similar to the original baicalein. Moreover, the 8-bromobaicalein effectively inhibited DENV1-4, and ZIKV viruses at the EC_50_s of 0.66–0.88 μM (Table 2, Supplementary Fig. S3), suggesting a broad spectrum inhibition against mosquito-borne viruses. The EC_50_ of 8-bromobaicalein in DENV2 (Table 2) was 4.59 times more effective than the original baicalein (EC_50_ of 4.04 ± 1.37 µM, Supplementary Fig. S4, supporting the hypothesis that halogenation could potentiate the compound efficacies. However, the 8-bromobaicalein did not inhibit a nonenveloped enterovirus-A71 (Supplementary Fig. S2). The compound might inhibit influenza A and herpes simplex viruses similar to baicalein, with the efficacies that remain under investigation.

The computational study suggested that the most likely target was the NS5/RdRp (Figs. 2, 3), and the bromine atom contributed to an additional interaction with the residues in the pocket. The targets of flavones were previously reported at NS3 protease^42^, and NS5 RdRp^43^ using in silico and enzyme-based methods. Our molecular docking and MD simulation results supported the NS5 RdRp and NS5 MTase, but not the NS3 protease (Fig. 2A). However, subsequent RMSD, QSAR, and in vitro enzymatic assays excluded the MTase from the potential targets (Figs. 3, 4). The in vitro RdRp assay confirm the findings with the IC_50_ of 34.85 µM (Fig. 4B), which is around 7 times more efficient than the original baicalein with the IC_50_ of 241.53 µM (Fig. 4C). The 8-bromobaicalein was more potent in cells suspecting that the compound could have additional cellular targets. Flavonoids are well-known antioxidants^44^ which generally protect the cells against the DENV-induced ER oxidative stress^45^. The discrepancies between enzyme- and cell-based efficacies were also found in another report describing an RdRp inhibitor^46^. Therefore, we concluded that the flaviviral RdRp could be a target of 8-bromobaicalein.

Moreover, the compound also inhibited chikungunya virus (CHIKV) with EC_50_ of 0.49 ± 0.11 µM and the pandocking result revealed a potential target was the nsp1 methyltransferase (Van Cao et. al. manuscript in preparation). The CHIKV alphavirus belongs to the family *Togaviridae*, evolutionarily distinctive from the flaviviruses (DENV and ZIKV) of the family Flaviviridae. Therefore, it is likely that the potential target of the alphavirus would be different from those of flaviviruses.

Baicalein was previously reported as dengue, Zika, and Japanese encephalitis viruses inhibitor at EC_50_s of 0.004–24 µM, depending the assay and detection systems^32,47,48^ and the mechanism of action was proposed at the late stage suggesting the flaviviral replication. We observed similar results as the 8-bromobaicalein was still effective when introduced to virus-infected cells as late as 12 h after infection (Fig. 5). Moreover, the 8-bromobaicalein inhibited the DENV3 and ZIKV RNAs when added 1, 6, and 12 h after infection (Supplementary Fig. S8). The animal showed mild toxicity from a slight ALT increase in the adult cohort suspecting a drug-induced hepatitis (Fig. 6C). The effect was completely reversible at the 3rd and the 8th days^49^. This finding was similar to brominated pinostrobin and pinocembrin^40^, which exhibited a slight increase in ALT levels on the first day after drug administration. Interestingly, the infant cohort showed higher tolerance to the compound as ALT and Cr levels were similar to the vehicle controls (Fig. 6A,B). The differential function of the cytochrome P450 1A2, 2D6, 2C19, or 3A4 were previously reported to metabolize flavonoid aglycones^50^. Generally, cytochrome P450 (CYP) pathways metabolize the flavonoids and a metabolized form is excreted in urine or bile. In contrast, the flavonoids primarily excreted by renal clearance remained an original form in urine^51^. Moreover, the cytochrome P450 were differentially expressed in fetal, infant, and juvenile livers^52^. Mainly, the cynomolgus macaque CYPs (mfCYPs) in the CYP1-4 subfamilies were much less abundant in fetus but increasing rapidly after birth. It could be possible that the major route of excretion were different in infant and adult as renal clearance and CYP metabolism, respectively. Further investigation should include the analysis of compound species in urine.

In summary, this article reported a chemically modified natural product 8-bromobaicalein as a potential inhibitor of dengue, and Zika viruses. The potential target was at the conserved RdRp pocket of the flavivirus. The compound also showed mild toxicity in infant and adult mice despite seven consecutive days of high-dose administrations.

## Materials and methods

### Synthesis and identification of 8-bromobaicalein

8-Bromobaicalein was synthesized in a two-step process (Fig. 1A), including hydrolysis of baicalin^25^ (a) and bromination of baicalein^26^ (b). Briefly, the hydrolysis was conducted by addition of 98% H_2_SO_4_ (2.0 mL, 0.037 mmol) dropwise into baicalin (50 mg, 0.11 mmol) at room temperature with continuous stirring followed by slow addition of water (2.0 mL). After the evolution of heat terminated, one portion of water (15 mL) was poured directly into the mixture, and the yellow powder was collected by suction filtration. The bromination was performed by mixing 35 mg (0.13 mmol) baicalein and 33 mg (0.19 mmol) N-Bromosuccinimide (NBS) in 4.0 mL tetrahydrofuran (THF) in the presence of 5.0 *µ*L concentrated H_2_SO_4_. The reaction mixture was stirred at room temperature for 12 h before extraction with EtOAc. The precipitated product was washed with 10% aqueous NaHSO_4_ solution, dried over anhydrous Na_2_SO_4_, and concentrated under reduced pressure. The residue was recrystallized from MeOH to the target compound (35%) as a yellow powder. The brominated baicalein was identified by ^1^H NMR, δ (CD_3_OD, ppm) 8.04 (m, 2H), 7.56 (m, 3H), 6.77 (s, 1H)^26^ (Fig. 1B).

### Antiviral efficacy and cellular toxicity

LLC/MK2 (ATCC^®^ CCL-7), HEK-293 (ATCC^®^ CRL-1573), HepG2 (ATCC^®^ HB-8065), THP-1 (ATCC^®^ TIB-202), Huh-7, RD (ATCC^®^ CCL-136™), and C6/36 (ATCC^®^ CRL-1660) cell lines were propagated and maintained as previously described^16,21,40,53^. Reference DENV1 (16007), DENV2 (NGC, 16681), DENV3 (16562), DENV4 (1036), ZIKV (SV0127/14), and EV-A71 (BRCR) were propagated in Vero, C6/36, and R.D. cells as described^16,21^ Phumee et al*.*^53^. Cytotoxic concentration (CC_50_) of LLC/MK2, THP-1, HEK-293, HepG2, Huh-7, and R.D. cells were analyzed as previously described^40^. Effective concentration (EC_50_) of the compound against the DENV1-4, ZIKV, and CHIKV viruses were tested using LLC/MK2 cells^16,21^, whereas EV-A71 virus was tested using R.D. cells^54^, (https://www.mdpi.com/1420-3049/27/6/1908). Briefly, cells were seeded overnight and infected with each virus at the multiplicity of infection (M.O.I.) of 0.1 for 1 h. The compound was added during and after infection, and cells were incubated for 72 h. Supernatants were collected for 96-well^55^ or a traditional 24-well plaque titration. EC50 results were means and standard errors of three independent experiments.

### Cell-based time-of- addition study

The protocol was adapted from a previous description^21^. Briefly, LLC/MK2 cells (5 × 10^4^) were seeded in a 24-well plate and incubated as described. Cells were infected with DENV2 NGC, unless otherwise indicated, at the M.O.I. of 0.1 before adding the compound at designated concentrations. DMSO at 1% was used as no inhibition control. Supernatants were collected to determined viral titers by plaque titration assay or RT-qPCR.

### Target identification by in silico study

#### Molecular docking between baicaleins and DENV proteins

The 3D structures of 8-bromobaicalein, baicalein, and native inhibitor of each target protein were constructed by GaussView 6 and structurally optimized by HF/6-31G(d) level of the theory using Gaussian 16^56^. Each ligand was docked into the four binding sites on E protein (PDB code: 1OKE) suggested by the previous study^57^, the allosteric site of NS2B/NS3 (PDB code: 3U1I)^58,59^, the SAM binding site of NS5/MTase (PDB code: 6KR2)^60^, and the native inhibitor (NITD-107) binding region of NS5/RdRp (PDB code: 3VWS)^61^. Note that the crystal structure of NS5/RdRp in complex with NITD-107 inhibitor derived from DENV3. In this study, the DENV2 NS5/RdRp structure was prepared by homology modelling implemented in the swissmodel web server^62^ by using DENV3 NS5/RdRp (PDB code: 3VWS) as a template structure. The native inhibitors for E protein, NS2B/NS3 protease, NS5/MTase, and NS5/RdRp are 3-100-22^63^, SYC-1307^64^, Sinefungine^65^, and NITD-107^61^, respectively. According to the standard procedure, the binding energy and binding pose were predicted using Autodock VinaXB^66^. Then, the structure of target proteins and ligands were converted to the required PDBQT format using AutoDockTools^67^. The conformer with preferential binding, or the lowest binding energy for each ligand–protein complex, was chosen as the initial complex structure to perform the molecular dynamics simulation.

#### Molecular dynamics simulation

The selected complex structures were carried out by MD simulation to distinguish preferential targets for our compounds. The protonation state of the 8-bromobaicalein and baicalein was determined at neutral pH by pKa calculation using Marvin 21.4.0, ChemAxon (https://www.chemaxon.com). Each ligand's partial charge was prepared following the standard protocol, whereas the other parameters were from the general AMBER force field^68^. The AMBER ff14SB force field was applied on the NS5/RdRp. The addition of hydrogen atoms was created by using the LEaP module implemented in AMBER20^69^. The TIP3P water model was used to solvate each system with a minimum distance of 10 Å from the protein surface. The ions were then randomly added to neutralize the simulated systems. Each complex system was performed under the periodic boundary condition (P.B.C.) with the isothermal–isobaric (*N.P.T.*) scheme. The temperature gradually increased to 300 K for 100 ps, then continued at 300 K until 200 ns. Ligand stability was compared by analyzing the Root Mean Square Displacement (RMSD) of ligand, atomic contacts (#Atom), and the number of hydrogen bonding interactions (#Hbonds) between ligand and binding residues. The last 50-ns trajectories were used to calculate the number of compound-protein hydrogen bonds (H-bonds), the binding free energy ($\Delta {\text{G}}_{{{\text{bind}}}}$), and per-residue decomposition free energy ($\Delta {\text{G}}_{{{\text{bind}}}}^{{{\text{residue}}}}$) based on molecular mechanics/generalized Born surface area (MM/GBSA) method^70^. Only the amino acid residues exhibiting $\Delta {\text{G}}_{{{\text{bind}}}}^{{{\text{residue}}}}$ values lower than − 1.0 kcal/mol were considered for discussion.

### In vitro RNA-dependent RNA polymerase experiment

#### Expression and purification of DENV-2 RdRp

The production of DENV2 RdRp was performed according to a described protocol^29^. In brief, N-terminally Histidine-tagged DENV2 RdRp (N_His_-RdRp) was expressed with 200 µM IPTG (isopropyl-b-D-thiogalactopyranoside) induction, at 37 °C for 4 h. Cell disruption and clarification were sequentially done via sonication and centrifugation at 15,000 rpm for 1 h at 4 °C. His-tagged RdRp was purified by immobilized metal affinity chromatography (IMAC) with 175 mM imidazole elution. His-tag was removed by adding 2 units thrombin per 1 mg protein during dialysis for 2 days at 4 °C. The non-tagged RdRp was further purified by size exclusion chromatography. The purified enzyme was verified by Coomassie staining and Western blotting with anti-NS5 PA5-32200 (Thermo Fisher Scientific, Waltham, MA, USA) and anti-6 × His ab1187(Abcam, Cambridge, UK) antibodies.

#### Determination of RdRp activity (IC_50_)

The RdRp assay for inhibition testing was adapted from the previous publications^46,71^. Each reaction was performed in 25 µl reaction using the RdRp from DENV-2. 20 µl mixture of 1.25 µM RdRp and various concentrations of the 8-bromobaicalein and baicalein compounds in the reaction buffer (25 mM Tris–HCl pH7.5, 3 mM MnCl_2_, and 4 mM DTT) was incubated at 30 °C for 30 min. Subsequently, 5 µl initiator containing 5 mM rATP and 2.5 µM poly-U dissolved in the reaction buffer was added and incubated at 30 °C for 120 min. The final concentrations of the reaction components are 1 µM RdRp, 0.5 µM poly-U, 1 mM rATP, 25 mM Tris–HCl (pH 7.5), 13.3 mM KCl, 1% glycerol, 3 mM MnCl_2_, 4 mM DTT, 1% DMSO, and various concentrations of TH024 compound. After incubating at 30 °C for 2 h, 10 µl of stop solution containing 25 mM Tris–HCl (pH 7.5) and 105 mM EDTA was added to terminate the reaction. The nascent double-stranded RNA was quantitated by mixing 10 µl of the reaction mixture with 10 µl of 5 µM SYTO-9 dye in Tris–EDTA buffer and incubating at room temperature for 5 min. Fluorescence was measured at excitation and emission wavelengths of 485 nm and 520 nm, respectively, by using EnSight Multimode Plate Reader (Perkin Elmer, Waltham, MA, USA). The IC_50_ value was calculated from three independent experiments by using PRISM version 9 (GraphPad Software, Inc., La Jolla, CA, USA).

### Methyltransferase assay

#### Expression and purification of DENV-2 MTase

The production of DENV2 MTase was performed according to a described protocol^30^. In brief, N-terminally Histidine-tagged DENV-2 MTase (N_His_-MTase) synthesized according to previous description^30^ (GenScript; Piscataway, NJ, USA) was expressed with 100 µM IPTG (isopropyl-b-D-thiogalactopyranoside) induction, at 27 °C for 16 h. Cell disruption and clarification were sequentially done via sonication and centrifugation at 12,000 rpm for 30 min at 4 °C. His-tagged RdRp was purified by immobilized metal affinity chromatography (IMAC; Cytiva, Marlborough, MA, USA) with 500 mM imidazole elution. The eluate was dialyzed against 2 L of 50 mM Tris, pH 7.5, 50 mM NaCl, 1 mM MgCl_2_, 40% glycerol, and 1 mM DTT at 4 °C overnight. The purified enzyme was verified by Coomassie staining and methyltransferase activity.

#### Preparation of a 5’-capped RNA substrate substrate

A short nucleotide DNA template containing T7 and 5’UTR of DENV was amplified from the plasmid^30^. The 50 µl reaction mixture contained 5 µl 10 × Taq buffer with KCl, 10 µM each primer (final concentration of 0.2 µM), 10 mM dNTP mixture (final concentration of 0.2 mM), 3 µl 25 mM MgCl_2_, Taq DNA polymerase (1.25 U/reaction) and 10 ng plasmid template. The condition for PCR are initial denaturation at 95 °C for 3 min, followed by 30 cycles of denaturation at 95 °C for 30 s, annealing at 53 °C for 30 s and extension at 72 °C for 20 s, and final extension at 10 min. The PCR product was verified by agarose gel electrophoresis and purified using NucleoSpin^®^ Gel and PCR Clean-up (MACHEREY–NAGEL, Düren, Germany).

The in vitro transcription of the short nucleotides was performed to produced 200 nucleotides RNA using MEGAscript™ T7 Transcription Kit (Invitrogen, Waltham, MA, USA) according to the manufacturer’s protocol. The 20 µl reaction mixture contained of 2 µl 10 × reaction buffer, 2 µl each NTP solution, 2 µl T7 enzyme mix and 0.1 µg DNA template. The reaction mixture was incubated at 37 °C for 6 h. 1 µl TURBO DNase (2 U/µl) was added and incubated at 37 °C for 15 min to digest the DNA template. The RNA was purified using NucleoZOL (MACHEREY–NAGEL, Düren, Germany) and dissolved in RNase-free water. The RNA integrity was verified by agarose gel electrophoresis and stored in aliquoted at − 80 °C.

The 5’-capped RNA substrate was prepared from 200 nucleotides RNA using Vaccinia capping system (New England Biolabs, Ipswich, MA, USA) according to the manufacturer’s protocol. 15 µl RNA (10 µg) was incubated at 65 °C for 5 min, then placed on ice for 5 min. The 20 µl reaction mixture contained denatured RNA, 2 µl 10 × capping buffer, 1 µl 10 mM GTP, and 1 µl vaccinia capping enzyme. The reaction mixture was incubated at 37 °C for 30 min. The RNA was purified using NucleoZOL and dissolved in RNase-free water.

#### Determination of methyltransferase activity (IC_50_)

The MTase assay for inhibition testing was adapted from the previous publications^30^. The MTase assay for inhibition testing was performed using MTase-Glo™ Methyltransferase Assay according to the manufacturer’s protocol (Promega, Madison, Wisconsin, USA). Each reaction was performed in 50 µl reaction using the MTase from DENV-2. 10 µl enzyme complex (100 µg/ml MTase, 1.25 µM capped RNA substrate and 10 × MTase-Glo reagent in the 4X reaction buffer (80 mM Tris–HCl pH 8.0, 200 mM NaCl, 4 mM EDTA, 12 mM MgCl2, 0.4 mg/ml BSA and 4 mM DTT)) was mixed with 5 µl various concentration of 8-bromobaicalein and incubated at room temperature for 10 min. Subsequently, 10 µl of 2.5 µM S-adenosylmethionine (SAM) was added and incubated at 37 °C for 10 min. The final concentrations of the reaction components are 200 ng MTase, 0.5 µM capped RNA, 1 µM SAM, 20 mM Tris–HCl pH 8.0, 50 mM NaCl, 1 mM EDTA, 3 mM MgCl_2_, 0.1 mg/ml BSA and 1 mM DTT, 2% DMSO, and various concentrations of 8-bromobaicalein compound. After incubating, 25 µl of MTase-Glo detection solution was added and incubated at room temperature for 60 min. The luminescence was measured using VICTORTM X3 2030 Multilabel Reader (Perkin Elmer, Waltham, MA, USA). The percent enzyme activity was calculated from two independent experiments by using PRISM version 9 (GraphPad Software, La Jolla, CA, USA).

### Animal toxicity

All methods were carried out in accordance with relevant guidelines and regulations. All experimental protocols were approved by the Institutional Animal Care and Use Committee of the Faculty of Medicine, Chulalongkorn University, Bangkok, Thailand (certificate number: 027/2562), based on the National Institutes of Health, U.S.A.'s criteria for the use and treatment of laboratory animals and by the National University of Singapore (protocol number: R20-0073). All methods are reported in accordance with ARRIVE guidelines (https://arriveguidelines.org) for the reporting of animal experiments. The 8-bromobaicalein was daily administered at 10 mg/kg via intraperitoneal injection for 7 days (n = 6) to determine the drug safety. The control group (n = 6) was injected with the vehicle (5% DMSO, 33.25% polyethylene glycol (PEG.)-400, 1.90% ethanol, and 59.85% distilled water). All mice were weighed daily and monitored for clinical signs using a clinical scoring system. Blood samples were collected via submandibular and cardiac puncture at the 8th day after the first compound administration for alanine transaminase (EnzyChrom, EALT-100, BioAssay, Hayward, CA, USA), and serum creatinine (QuantiChromTM, DICT-500, BioAssay, Hayward, CA, USA) as markers of hepatotoxicity and nephrotoxicity, respectively.

## Supplementary Information

Supplementary Figures.

## Data Availability

DENV1 (16007), DENV2 (NGC, 16681), DENV3 (16562), DENV4 (1036), ZIKV (SV0127/14), and EV-A71 (BrCr) sequence data were available in Genbank nucleotide (https://www.ncbi.nlm.nih.gov/nuccore) under the following accession numbers; MW945844.1, NC_001474.2, KU725663.1, KU725665.1, MW945703.1, MG770188.1, U22521.1. The DENV2 RdRp protein (P29990) in the enzyme study can be found in uniprotKB (https://www.uniprot.org/uniprot/P29990#PRO_0000037935). The DENV2 MTase protein (1L9K) in the enzyme study can be found in PDB (https://www.rcsb.org/structure/1l9k). The dengue protein in the computational study; dimeric envelope protein in complex with n-octyl-beta-D-glucoside (10KE), NS2B/NS3 protease (3U11), NS5/MTase (6KR2), and NS5/RdRp bound to NITD-107 (3VWS), and the compound structure of known inhibitors; 3-100-22 56, SYC-130757, Sinefungine 58, and NITD-107, are available in the RCSB protein data bank (https://www.rcsb.org/). AutoDock VinaXB (https://vina.scripps.edu/) was used for virtual screening by molecular docking technique. The molecular visualization and compound structural construction were performed using Chimera USF (https://www.cgl.ucsf.edu/chimera/) and VMD 1.9.3 (https://www.ks.uiuc.edu/Risearch/vmd/), which is free for academic users. Gnuplot (http://www.gnuplot.info) and adobe illustration 25.4.1 (https://www.adobe.com/products/illustrator.html) were used for plotting data and graphical visualization. Scripts for data analysis and others are available from the authors upon request.
