# Author Correction: The 8-bromobaicalein inhibited the replication of dengue, and Zika viruses and targeted the dengue polymerase

**DOI:** 10.1038/s41598-023-34883-5

**Published:** 2023-05-11

**Authors:** Siwaporn Boonyasuppayakorn, Thanaphon Saelee, Thao Nguyen Thanh Huynh, Rita Hairani, Kowit Hengphasatporn, Naphat Loeanurit, Van Cao, Vipanee Vibulakhaophan, Panattida Siripitakpong, Parveen Kaur, Justin Jang Hann Chu, Chairat Tunghirun, Opas Choksupmanee, Sarin Chimnaronk, Yasuteru Shigeta, Thanyada Rungrotmongkol, Warinthorn Chavasiri

**Affiliations:** 1 Center of Excellence in Applied Medical Virology, Department of Microbiology, Faculty of Medicine, Chulalongkorn University, Bangkok, 10330 Thailand; 2 Center of Excellence in Natural Products Chemistry, Department of Chemistry, Faculty of Science, Chulalongkorn University, Bangkok, 10330 Thailand; 3 Center for Computational Sciences, University of Tsukuba, 1-1-1 Tennodai, Tsukuba, Ibaraki 305-8577 Japan; 4 Graduate School, Interdisciplinary Program in Microbiology, Chulalongkorn University, Bangkok, 10330 Thailand; 5 Department of Biology, Faculty of Science, Chulalongkorn University, Bangkok, 10330 Thailand; 6 Center of Excellence in Biocatalyst and Sustainable Biotechnology, Department of Biochemistry, Faculty of Science, Chulalongkorn University, Bangkok, 10330 Thailand; 7 Department of Microbiology and Immunology, Yong Loo Lin School of Medicine, National University of Singapore, Singapore, 117545 Singapore; 8 Infectious Diseases Translational Research Programme, Yong Loo Lin School of Medicine, National University of Singapore, Singapore, Singapore; 9 NUS Medicine BSL3 Core Facility, Yong Loo Lin School of Medicine, National University of Singapore, Singapore, Singapore; 10 Institute of Molecular and Cell Biology (IMCB), A*STAR, Singapore, Singapore; 11 The Laboratory of RNA Biology, Institute of Molecular Biosciences, Mahidol University, Salaya Campus, Nakhon Pathom, 73170 Thailand; 12 Program in Bioinformatics and Computational Biology, Faculty of Science, Chulalongkorn University, Bangkok, 10330 Thailand

Correction to: *Scientific Reports* 10.1038/s41598-023-32049-x, published online 25 March 2023

In the original version of this Article Sarin Chimnaronk was incorrectly affiliated with ‘Infectious Diseases Translational Research Programme, Yong Loo Lin School of Medicine, National University of Singapore, Singapore, Singapore.’ The correct affiliation is listed below.

The Laboratory of RNA Biology, Institute of Molecular Biosciences, Mahidol University, Salaya Campus, Nakhon Pathom, 73170, Thailand.

The original Article has been corrected.

