# Supplementary material for: The 8-bromobaicalein inhibited the replication of dengue, and Zika viruses and targeted the dengue polymerase
Source: Sci Rep. 2023 Mar 25;13:4891. doi: 10.1038/s41598-023-32049-x (PMC10039358; doi:10.1038/s41598-023-32049-x)
Supplement: Supplementary file 1 — Supplementary Figures. [file 41598_2023_32049_MOESM1_ESM.docx]

**Supplementary Figures**

**Supplementary Fig. S1.** 8-bromobaicalein (TH-024) identification by ^13^C-NMR (DMSO-*_d6_*): *δ*(ppm).

**Supplementary Fig. S2.** CC50s of 8-bromobaicalein (TH024) against various cell lines. Briefly, cells were seeded overnight before the compound addition. After 48 h of co-incubation, the cell viabilities were demonstrated with the MTS assay.

**Supplementary Fig. S3.** EC50s of 8-bromobaicalein (TH024) against viruses-infected LLC/MK2 cells.

**Supplementary Fig. S4.** EC_50_ and CC_50_ of baicalein against DENV2-infected LLC/MK2 cells.


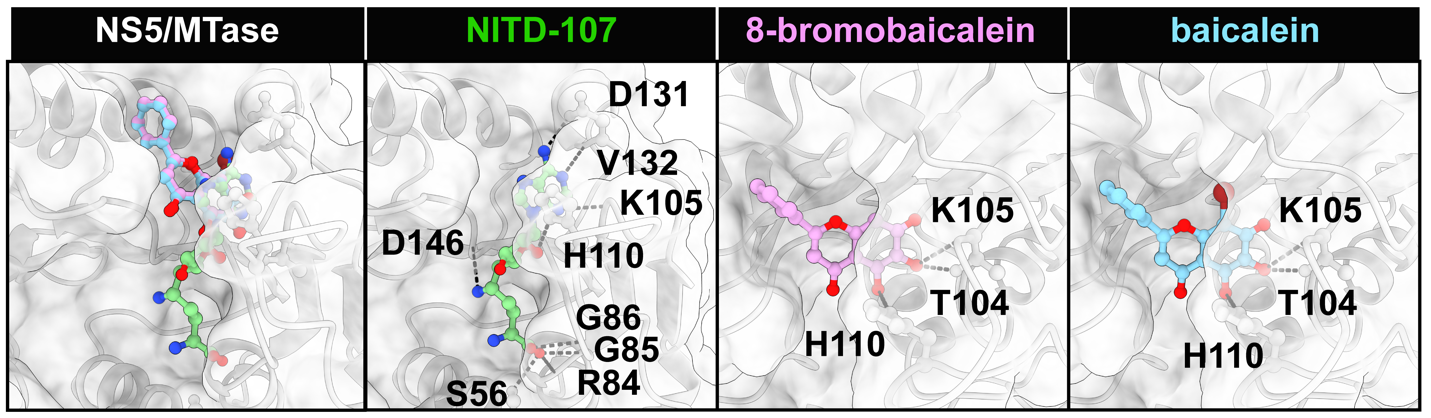


**Supplementary Fig. S5.** Binding conformation and hydrogen bonding interaction of Sinefungin, 8-bromobaicalein, and baicalein in complex with DENV NS5/MTase.


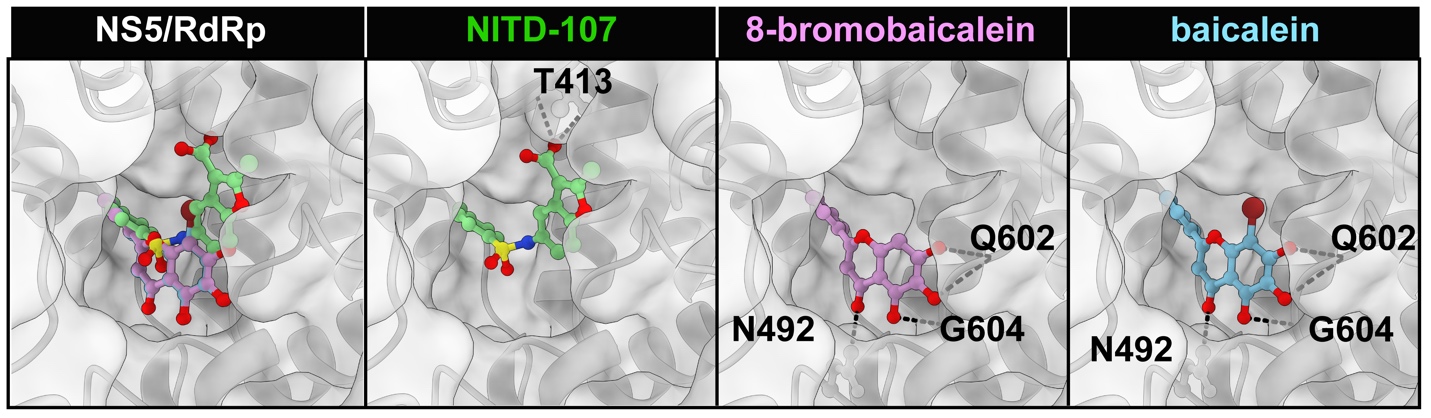


**Supplementary Fig. S6.** Binding conformation and hydrogen bonding interaction of NITD-107, 8-bromobaicalein, and baicalein in complex with DENV NS5/RdRp.

**
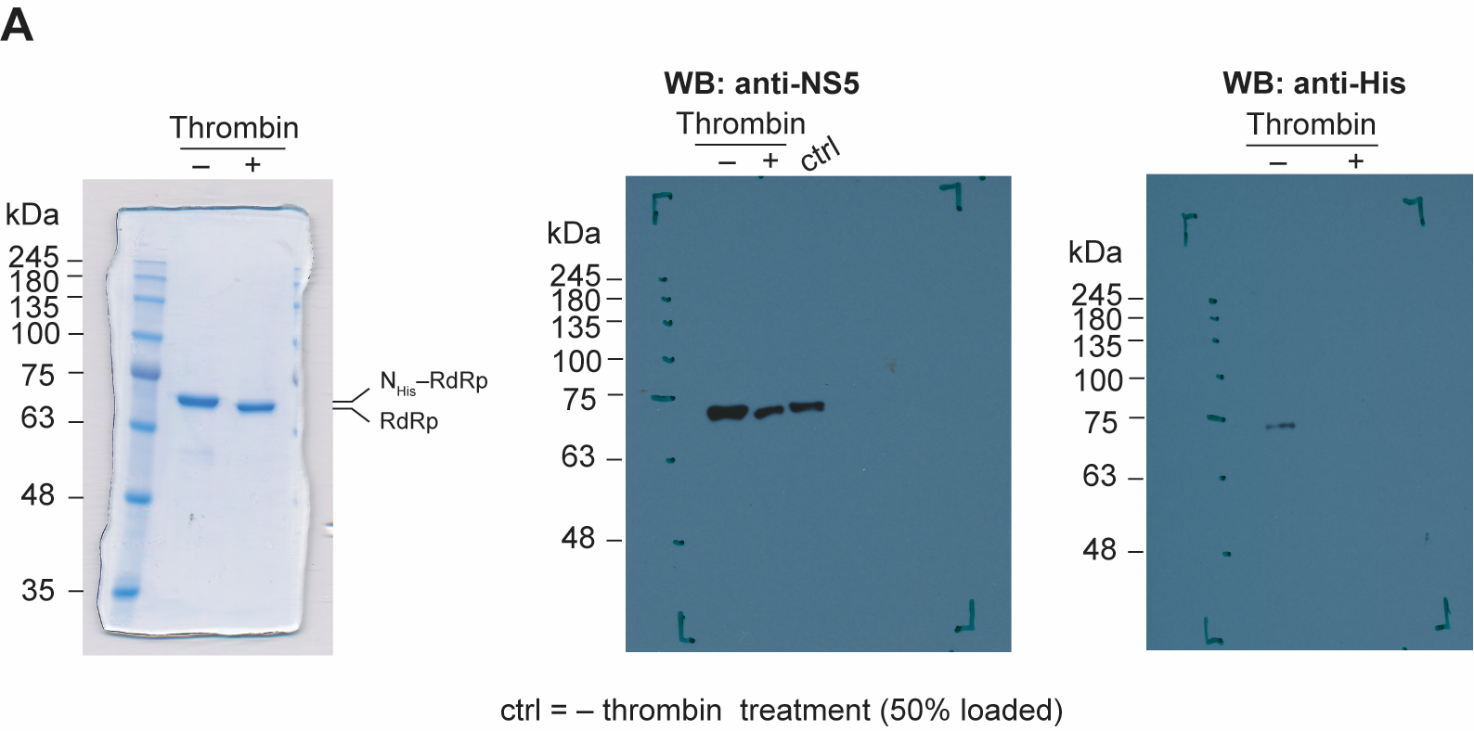
**


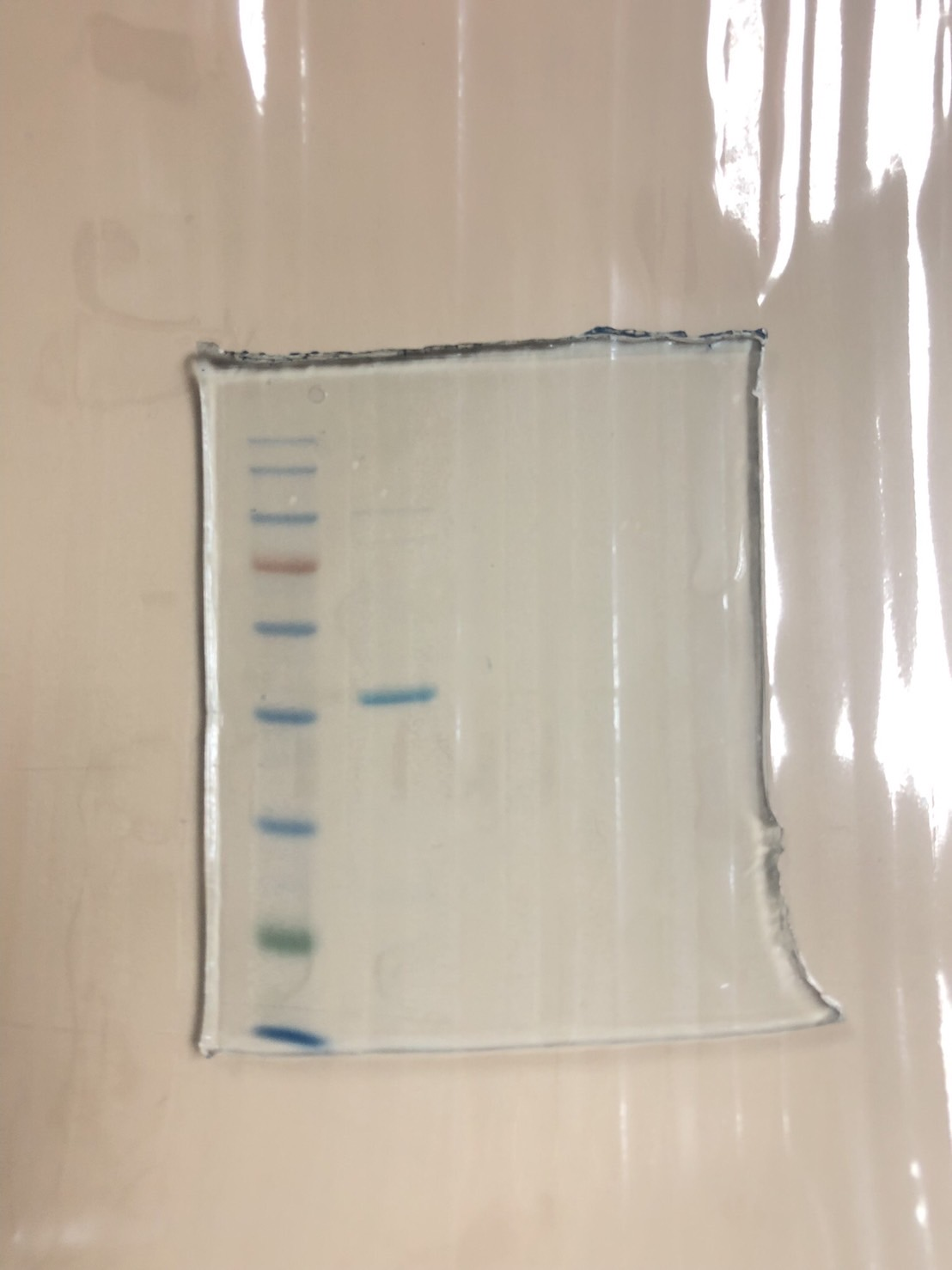


**Supplementary Fig. S7.** The full-length gels and blots **with membrane edges**of Fig. 4 A and 4D.

**Supplementary Fig. S8.** The time-of-addition of 8-bromobaicalein against DENV3-, and ZIKV-infected LLC/MK2 cells. Briefly, the 10 µM 8-bromobaicalein was added to the virus-infected cells (M.O.I. of 1) at 1, 6, 12, 24, and 48 h post-infection. Supernatants were collected at 48 h after infection for analysis by RT-qPCR. Results were reported as percent inhibition from DMSO-treated cells.
